# Depression and diabetic retinopathy: an underexplored connection

**DOI:** 10.3389/fnut.2025.1557105

**Published:** 2025-04-11

**Authors:** Jingwen Li, Hao Sun, Ling Li, Xuelin Xin, Xiao Zhang, Weihong Lv, Shanjuan Tan

**Affiliations:** ^1^Ophthalmology, Qingdao Municipal Hospital, Qingdao, China; ^2^Qingdao Municipal Hospital, University of Health and Rehabilitation Sciences, Qingdao, China; ^3^Department of Healthcare-Associated Infection Management, Qingdao Municipal Hospital, University of Health and Rehabilitation Sciences, Qingdao, China

**Keywords:** diabetic retinopathy, depression, risk factors, NHANES, mental health

## Abstract

**Background:**

Diabetic retinopathy, a major microvascular complication of diabetes, significantly contributes to global blindness. Emerging evidence suggests a potential association between depression and DR, yet the mechanisms remain unclear.

**Objectives:**

This study aimed to investigate the relationship between depression and DR using nationally representative data from the National Health and Nutrition Examination Survey (NHANES, 2011–2020).

**Methods:**

Depression was assessed using the PHQ-9, and DR was identified through self-reported diagnoses. Multivariable logistic regression and restricted cubic splines analyzed the relationship, adjusting for demographic and clinical covariates. Subgroup analyses examined interactions with factors like age and lipid control.

**Results:**

Among 1,653 participants, the weighted DR prevalence was 18.91%. Depression was independently associated with higher DR risk (OR: 1.69, 95% CI: 1.08–2.64, *p* < 0.05), with a significant linear relationship between PHQ-9 scores and DR (*p* < 0.01). Subgroup analyses highlighted stronger associations in older adults and those with well-controlled lipids.

**Conclusion:**

Depression is a significant and independent risk factor for DR, emphasizing the need for integrated management of mental health and metabolic control in diabetic patients. Addressing depression may reduce DR burden and improve overall quality of life.

## 1 Introduction

Diabetic retinopathy (DR) is one of the major microvascular complications of diabetes, primarily affecting the retina. It results from retinal vascular damage caused by prolonged hyperglycemia. DR is a leading cause of blindness among working-age populations worldwide (1). Numerous studies have shown that the global prevalence of diabetic retinopathy is steadily increasing, with an estimated one-third of diabetic patients being affected (2). Specifically, in 2019, the global number of diabetes patients reached 463 million, and this figure is expected to rise to 700 million by 2045 (3). Among these patients, the prevalence of diabetic retinopathy is approximately 34.6%, and in certain regions, particularly in China, the prevalence of diabetic retinopathy is as high as 34.08% (4).

Diabetic retinopathy poses a significant threat to public health. It not only remains a leading cause of adult blindness, severely impacting patients’ quality of life, but also brings a substantial economic burden by increasing healthcare costs, potentially leading to workforce loss and a decline in societal productivity (2). Furthermore, the distribution of diabetic retinopathy is uneven across regions, especially in developing countries and resource-limited areas, where the lack of effective screening and treatment options often results in more severe disease progression (5).

Depression, as a common mental health issue, is characterized by high prevalence, disability rates, and suicide risk (6). According to the World Health Organization, depression has become one of the leading causes of disability worldwide, imposing a significant economic burden on society. Depression not only severely impacts patients’ quality of life but is also closely linked to various physical conditions. In general hospitals, patients often present with comorbid anxiety and depressive symptoms, which may exacerbate disease progression and affect treatment outcomes (7). Therefore, it is particularly important to address the mental health of patients with chronic diseases (8).

Currently, studies have suggested a potential association between depression and diabetic retinopathy. A study based on multiple database analyses over an 18-year period found that depression may be a risk factor for diabetic retinopathy (9). Furthermore, prolonged psychological stress may promote the onset and progression of diabetic retinopathy through dysfunction of the neuroendocrine and immune systems (10).

Although existing research indicates a possible connection between depression and diabetic retinopathy, studies on the relationship between the two remain limited, and the specific mechanisms underlying this association remain unclear. Therefore, this study aims to investigate the relationship between depression and diabetic retinopathy by analyzing extensive data from the National Health and Nutrition Examination Survey (NHANES) conducted between 2011 and 2020. The goal is to fill this research gap and provide new insights and theoretical support for the field.

## 2 Materials and methods

### 2.1 Study design and sample

**Figure 1:**
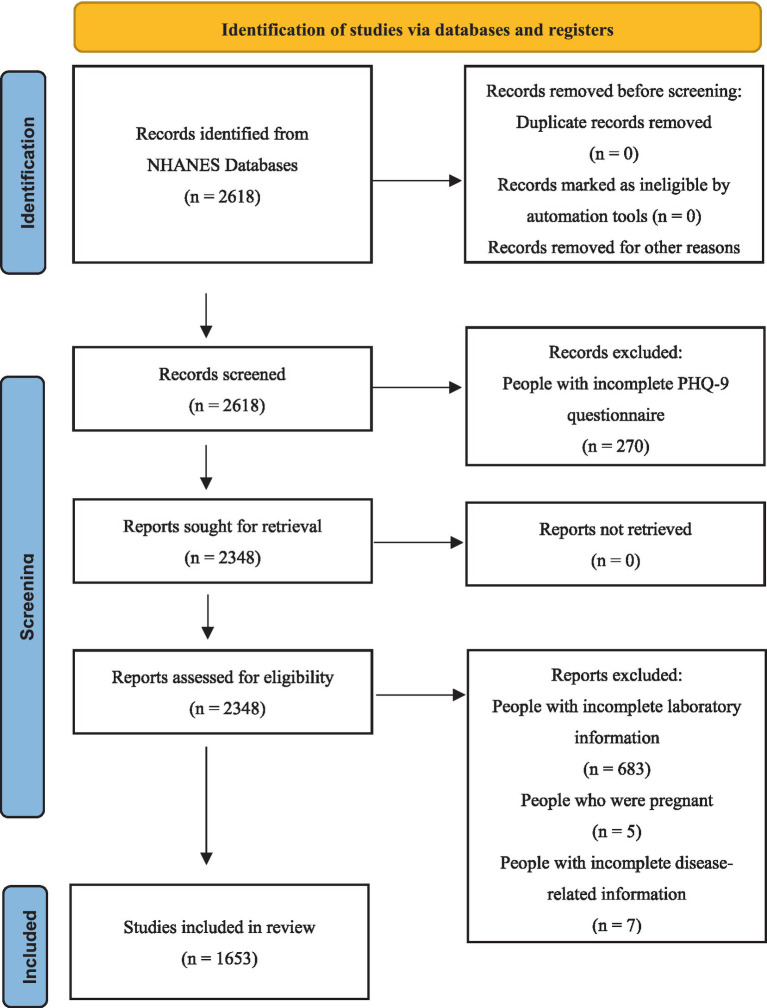
Flowchart for screening the study population.

The entire dataset of the study population was obtained from the NHANES database. All data from NHANES 2011–2020 were approved by the Institutional Review Board, and all participants provided informed consent and written documentation (11). Due to a low number of participants who completed the diabetes interview questionnaire, the initial cohort included 2,618 individuals. Among them, 270 participants were excluded due to incomplete PHQ-9 questionnaire assessments. Additionally, 683 subjects with incomplete laboratory test data were excluded. Among the remaining 1,665 participants, further exclusions were made, including 5 pregnant participants and 7 individuals with missing disease-related data. Ultimately, 1,653 participants met the eligibility criteria for the study. The screening process for the study population is shown in Figure 1.

### 2.2 Assessments

In this study, we used the Patient Health Questionnaire-9 (PHQ-9) to assess participants’ depressive symptoms. The PHQ-9 consists of nine items and is a widely used screening tool for depression, which measures the frequency of depressive symptoms over the past 2 weeks. Each item is scored on a scale from 0 to 3, with a total score ranging from 0 to 27. A total score of 10 or higher is considered indicative of depressive symptoms (12, 13).

In this study, DR was defined based on self-reported diagnoses confirmed by professional physicians, consistent with previous literature (14, 15). In the diabetes interview questionnaire, participants diagnosed with diabetes were asked, “Did diabetes affect your eyes/have retinopathy?” A positive response was considered indicative of self-reported DR. The question “What was your last A1C level?” was used to report participants’ HbA1c levels. An HbA1c level below 7% was considered to indicate good control (15).

Standardized questionnaires were used to collect data on participants’ age, gender (male, female), ethnicity (Mexican American, other Hispanic, non-Hispanic White, non-Hispanic Black, and other races), marital status (married/partnered, divorced/widowed/separated, unmarried), smoking status (never smoked, former smoker, current smoker), family income-to-poverty ratio (PIR) (≤1.30, 1.30–3.50, >3.50), and body mass index (BMI) (<25 and ≥ 25). We categorized behavioral habits based on smoking frequency, classifying participants into: never (smoked fewer than 100 cigarettes in their lifetime), former (smoked more than 100 cigarettes in their lifetime but no longer smoke), and current (smoked more than 100 cigarettes in their lifetime, occasionally or daily).

Laboratory data were derived from serum samples of HANES participants, which were processed and packaged in the Mobile Examination Center (MEC), stored at 2–8°C, and shipped to the Advanced Research Diagnostic Laboratory (ARDL) at the University of Minnesota, Minneapolis, Minnesota, for analysis. Detailed experimental procedures and standards can be found at https://wwwn.cdc.gov/Nchs/Nhanes/2017-2018/P_BIOPRO.htm. The definitions and standards for each variable are as follows:

The diagnostic criteria for hypertension are as follows: average blood pressure ≥ 140/90 mmHg. The calculation method for average blood pressure is as follows: (1) When the diastolic pressure reading is zero, the average diastolic pressure is not calculated; (2) If all diastolic pressure readings are zero, the average is zero; (3) If there is only one blood pressure reading, that reading is taken as the average; (4) If multiple blood pressure readings are available, the first reading is always excluded from the average. Lipid control is determined by non-high-density lipoprotein (non-HDL) cholesterol, calculated as the total cholesterol minus HDL cholesterol. A non-HDL level < 130 mg/dL is considered indicative of good lipid control (16). Diabetic nephropathy (DN) is diagnosed when the urine albumin/creatinine ratio is 30 mg/g or higher (17, 18).

### 2.3 Statistical analyses

In this study, multiple imputation was applied to handle randomly missing data to minimize potential biases and improve the robustness of the analysis. For non-randomly missing data, defined as missing values that showed systematic patterns associated with specific characteristics of the sample or variables, exclusion was performed to maintain the representativeness of the sample. This decision was made to avoid introducing biases due to the selective absence of key information. The filtered dataset was analyzed using R (v.4.4.3). Continuous variables were described using the mean (standard deviation), with group comparisons conducted via *t*-tests. Categorical variables were described using frequencies and percentages *n* (%), with group comparisons conducted using chi-square tests.

In sensitivity analyses, we employed binary logistic regression for multivariable analysis, using both depression prevalence and PHQ-9 scores as independent variables. Multiple sensitivity analyses were conducted by incrementally incorporating 11 covariates into the model to ensure the robustness of the findings. Additionally, we performed subgroup analyses and cross-validation to identify high-risk populations more precisely. Restricted cubic splines (RCS) with three knots were used to explore the nonlinear relationship between depression and the risk of DR. Further stratified analyses were conducted based on two covariates—age and lipid control—where interaction effects were observed. In these analyses, all 11 covariates were adjusted.

All analyses in this study accounted for the complex, multistage sampling design of NHANES and used appropriate weighting calculations. Weighted multivariable logistic regression was performed to ensure the representativeness of the results. A *p*-value of <0.05 was considered statistically significant throughout the study.

## 3 Result

### 3.1 Characteristics of the included population

This study included 1,653 NHANES participants, representing a weighted estimate of 11,530,665 individuals in the U.S. population. The overall weighted prevalence of DR was 18.91%. Table 1 outlines the baseline characteristics of the study population, revealing significant associations between DR and various factors. Specifically, patients with DR exhibited significantly higher levels of glycated hemoglobin (HbA1c) (*p* = 0.02) and lower poverty-to-income ratio (PIR) (*p* = 0.02). Additionally, the albumin-to-creatinine ratio was significantly elevated in the DR group (*p* = 0.01), and the prevalence of depression was notably higher in DR patients (*p* = 0.02). Furthermore, the prevalence of diabetic nephropathy (DN) was significantly higher among DR patients compared to those without DR (*p* < 0.01). Collectively, these findings indicate that DR is closely linked to mental health, poor glycemic control, renal dysfunction, and socioeconomic status, emphasizing the need for a holistic approach in future studies.

**Table 1:** Table of baseline characteristics of the population.

Characteristics	Diabetic retinopathy			
	Total	No	Yes	*p*-value
Total	1,653 (100.00)	1,311 (81.09)	342 (18.91)	
Age ~ years	60.69 (0.42)	60.97 (0.49)	59.51 (0.94)	0.18
Gender ~ %				0.16
Female	759 (45.22)	629 (46.53)	130 (39.65)	
Male	894 (54.78)	682 (53.47)	212 (60.35)	
Race ~ %				0.62
Mexican American	184 (5.58)	140 (5.31)	44 (6.74)	
Non-Hispanic Black	365 (9.30)	286 (9.00)	79 (10.57)	
Non-Hispanic White	692 (71.45)	563 (71.83)	129 (69.83)	
Other Hispanic	142 (3.96)	108 (3.90)	34 (4.24)	
Other Race	270 (9.71)	214 (9.96)	56 (8.62)	
Marital status ~ %				0.92
Married/Living with partner	1,093 (69.64)	875 (69.54)	218 (70.06)	
Never married	151 (8.74)	117 (8.89)	34 (8.11)	
Widowed/Divorced/Separated	409 (21.62)	319 (21.57)	90 (21.83)	
Family PIR	3.17 (0.06)	3.22 (0.06)	2.92 (0.12)	0.02
BMI ~ kg/m2				0.17
<25	1,458 (91.11)	1,157 (90.57)	301 (93.44)	
≥25	195 (8.89)	154 (9.43)	41 (6.56)	
Smoking behavior ~ %				0.70
Former	606 (37.94)	481 (37.28)	125 (40.75)	
Never	848 (49.91)	677 (50.33)	171 (48.10)	
Now	199 (12.16)	153 (12.39)	46 (11.15)	
Hypertension ~ %				0.19
No	401 (22.73)	325 (23.52)	76 (19.34)	
Yes	1,252 (77.27)	986 (76.48)	266 (80.66)	
HbA1c ~ %	7.21 (0.05)	7.12 (0.05)	7.59 (0.19)	0.02
HbA1c control ~ %				0.06
High	899 (51.25)	679 (49.19)	220 (60.11)	
Low	754 (48.75)	632 (50.81)	122 (39.89)	
Albumin creatinine ratio ~ mg/g	105.05 (13.56)	84.31 (14.05)	193.94 (39.53)	0.01
DN ~ %				<0.01
No	1,164 (74.01)	972 (76.80)	192 (62.08)	
Yes	489 (25.99)	339 (23.20)	150 (37.92)	
Non-HDL ~ mg/dL	127.87 (1.84)	127.83 (1.86)	128.04 (3.95)	0.96
Lipid control ~ %				0.41
High	652 (41.29)	516 (40.48)	136 (44.73)	
Low	1,001 (58.71)	795 (59.52)	206 (55.27)	
Depression ~ %				0.02
No	1,487 (89.40)	1,194 (90.46)	293 (84.86)	
Yes	166 (10.60)	117 (9.54)	49 (15.14)	

### 3.2 Relationship between depression and risk of diabetic retinopathy

The analysis of the relationship between depression and the risk of DR is presented in Table 2. When depression prevalence was included as an independent variable, it was positively associated with DR risk (*p* = 0.02, OR: 1.69, 95% CI: 1.08–2.64), and this association remained significant after adjusting for multiple covariates (*p* < 0.05). When PHQ-9 scores were used as the independent variable, the positive association became even more pronounced (*p* < 0.01, OR: 1.05, 95% CI: 1.01–1.09), and this relationship also persisted after adjustment for multiple variables (*p* < 0.01).

**Table 2:** Association between depression, PHQ-9 scores, and risk of DR.

Variable	Model*	OR (95% CI)	*p*-value
Depression	Crude	1.69 (1.08–2.64)	0.02
	Model 1	1.82 (1.13–2.94)	0.01
	Model 2	1.66 (1.01–2.71)	0.04
	Model 3	1.65 (1.01–2.71)	0.05
PHQ9-Score	Crude	1.05 (1.01–1.09)	<0.01
	Model 1	1.06 (1.02–1.10)	<0.01
	Model 2	1.05 (1.01–1.09)	<0.01
	Model 3	1.05 (1.01–1.09)	<0.01

* Crude is the original model; Model 1 was adjusted for age, sex, and race; Model 2 was adjusted for age, sex, race, marital status, BMI, PIR, and smoking habits; and Model 3 was adjusted for age, sex, race, marital status, BMI, PIR, smoking habits, hypertension, lipid control, diabetic nephropathy, and HbA1C status.

**Figure 2:**
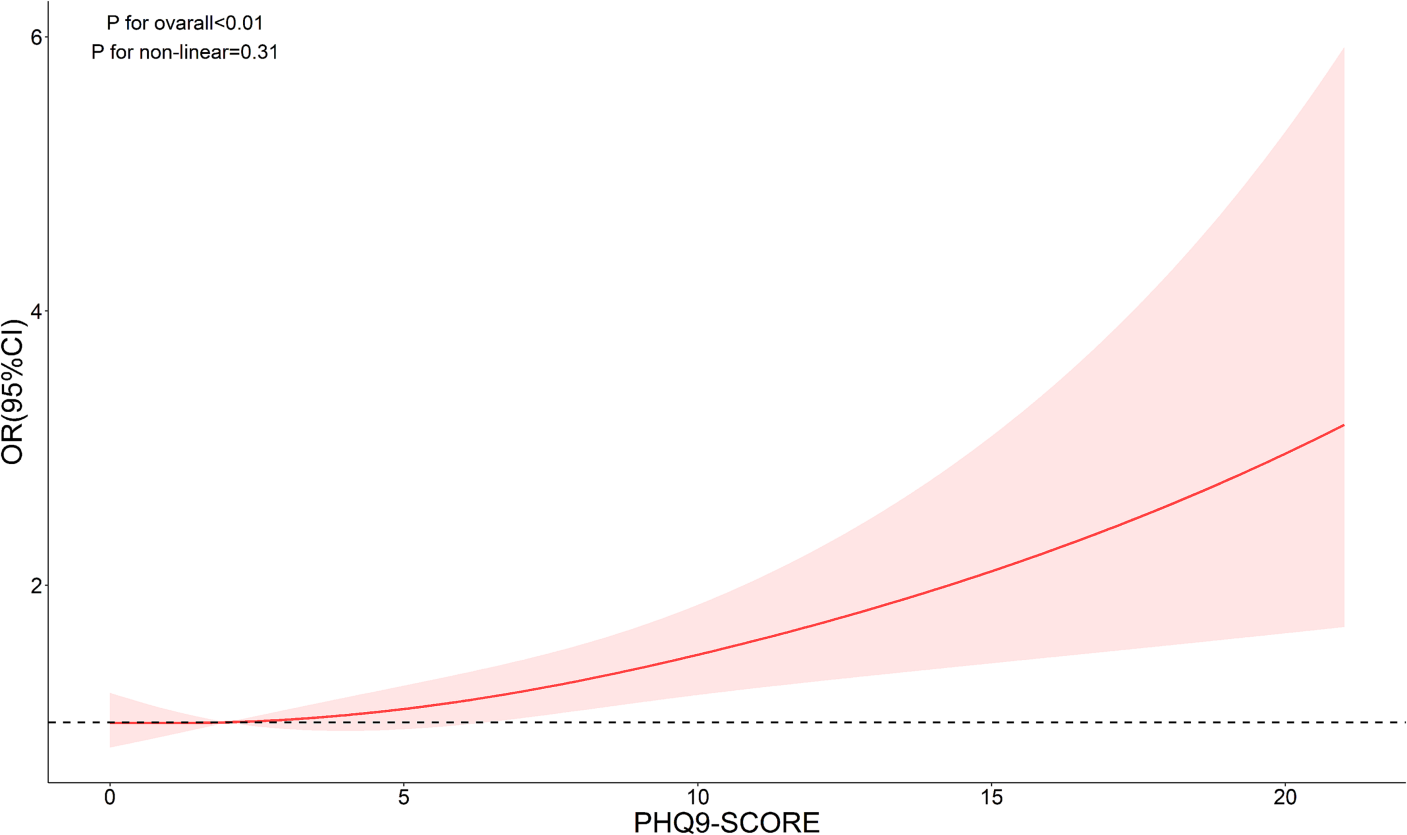
Restricted cubic spline curve (RCS) showing the relationship between PHQ-9 score and risk of DR.

**Figure 3:**
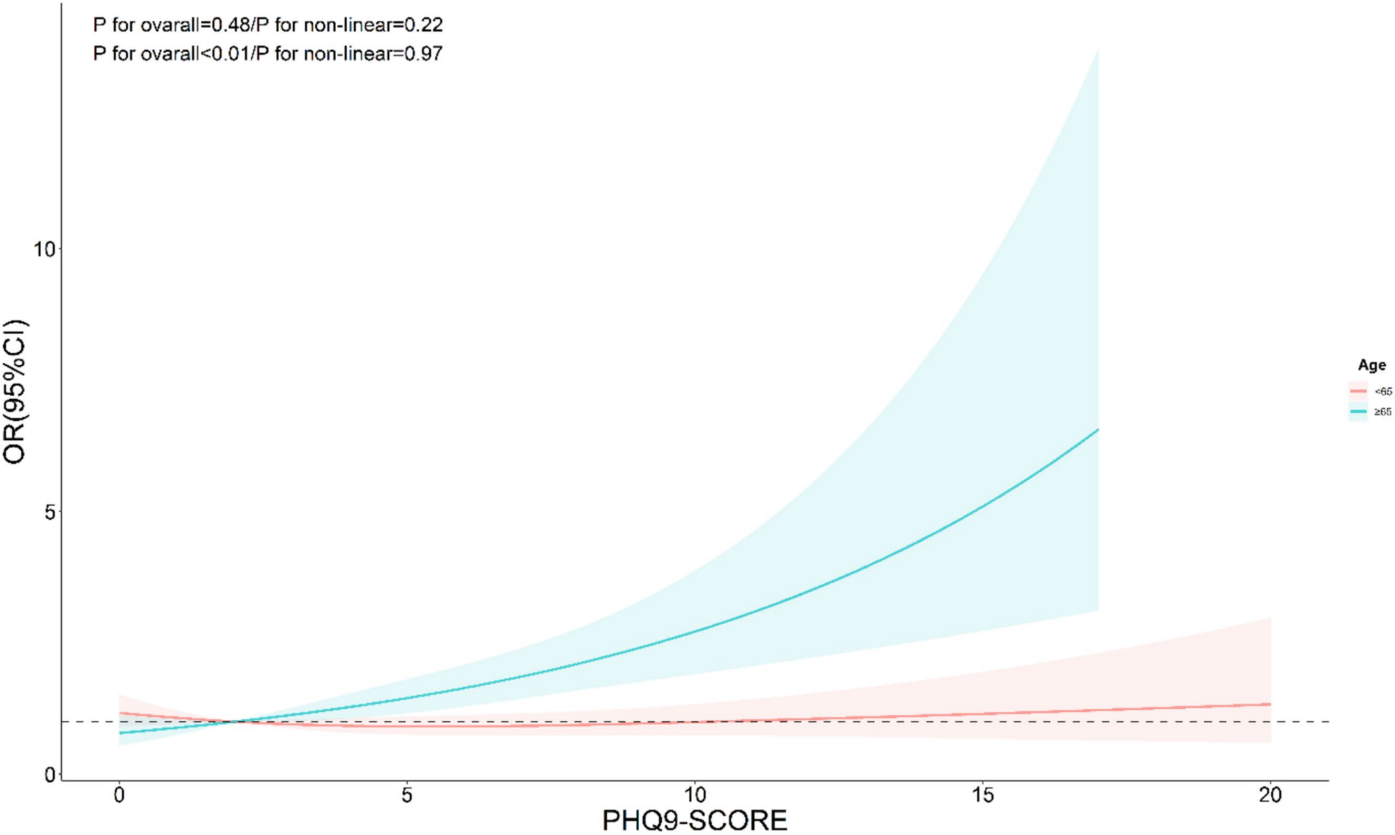
Restricted cubic spline curve (RCS) showing the relationship between PHQ-9 score and risk of DR, grouped by age.

**Figure 4:**
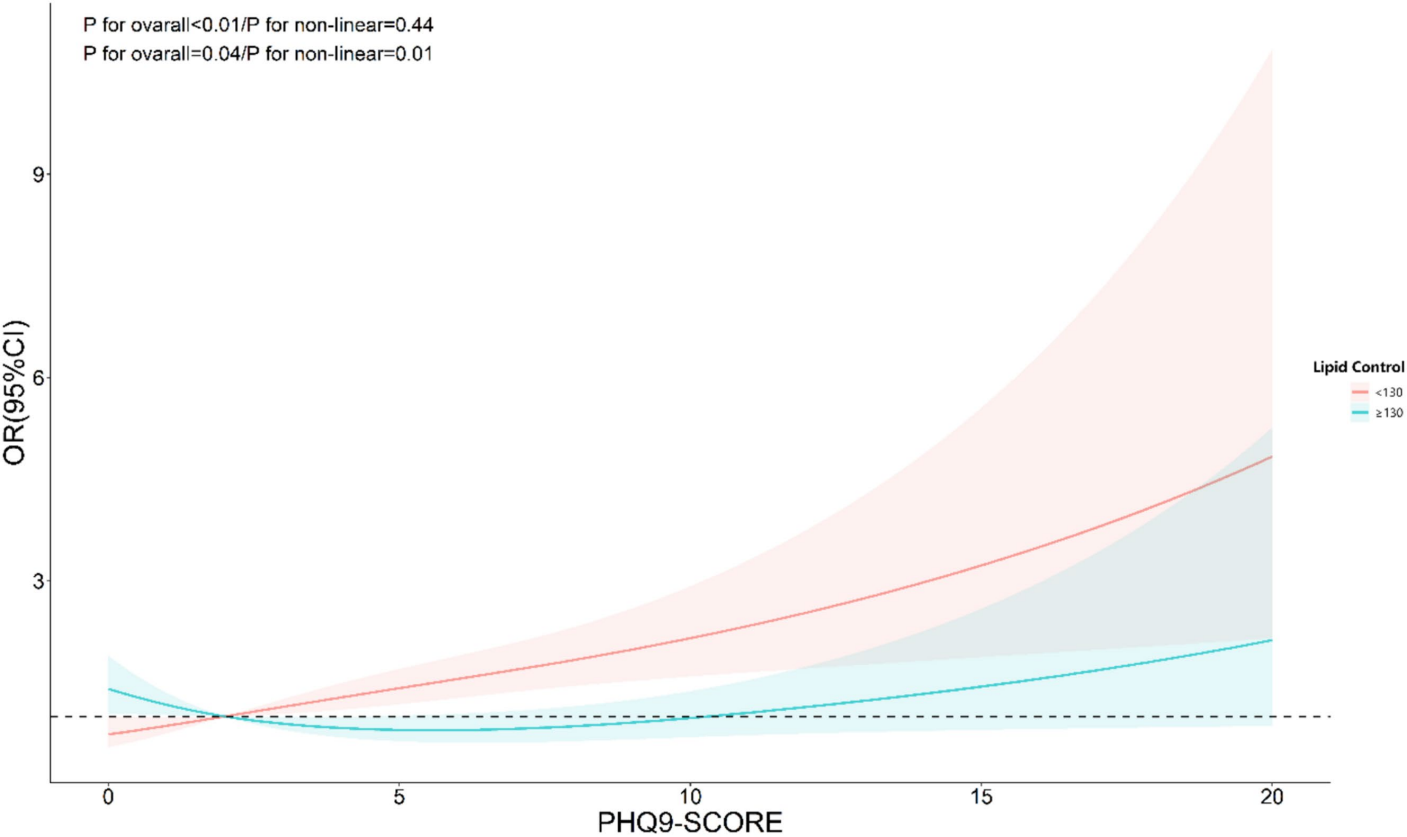
Restricted cubic spline curve (RCS) showing the relationship between PHQ-9 score and risk of DR, grouped by lipid control.

The nonlinear analysis of depression levels and DR risk, as shown in Figure 2, demonstrated a significant linear relationship after adjusting for all covariates (*p* for overall <0.01, *p* for non-linear = 0.31). Subgroup analyses based on age and lipid control status, illustrated in Figures 3, 4, revealed a significant positive association between depression levels and DR risk in older adults and individuals with well-controlled lipids (*p* for overall <0.01, *p* for non-linear >0.05). However, in individuals with poorly controlled lipids, a significant nonlinear relationship was observed (*p* for overall = 0.04, *p* for non-linear = 0.01).

### 3.3 Sub-group analysis

As shown in Table 3, subgroup analysis using depression as an independent variable revealed significant interactions between depression and age, sex, smoking status, and lipid control. The risk of DR was higher among individuals aged ≥65 years (*p* < 0.01), males (*p* < 0.01), former smokers (*p* < 0.01), and those with better lipid control (*p* = 0.01). Similarly, as indicated in Table 4, subgroup analysis with PHQ-9 scores as the independent variable showed that age and lipid control continued to exhibit significant interactions with depression scores. Higher depression scores were associated with an increased DR risk in individuals aged ≥65 years and those with better lipid control (*p* < 0.01). Consequently, stratified analyses were conducted in the investigation of nonlinear relationships between these factors. Additionally, although BMI also demonstrated a significant interaction with depression scores, the positive correlation between depression and DR risk remained consistent across both BMI subgroups. Notably, the risk was higher among individuals with a BMI <25 (OR: 1.40, 95% CI: 1.11–1.76).

**Table 3:** Results of subgroup analyses of depression and DR risk.

Subgroup variable	Diabetic retinopathy*		
	OR (95% CI)	*p* value	*p* for interaction
Age			0.02
<65	0.99 (0.54, 1.84)	0.98	
≥65	4.02 (1.77, 9.11)	<0.01	
Gender			<0.01
Female	0.55 (0.22, 1.41)	0.21	
Male	4.71 (2.03, 10.93)	<0.01	
Race			0.26
Mexican American	1.32 (0.30, 5.85)	0.67	
Non-Hispanic Black	1.45 (0.27, 7.81)	0.66	
Non-Hispanic White	1.91 (1.00, 3.64)	0.05	
Other Hispanic	0.27 (0.01, 10.14)	0.41	
Other Race	1.43 (0.54, 3.79)	0.46	
Marital status			0.41
Married/Living with partner	2.12 (1.17, 3.82)	0.01	
Never married	2.88 (0.36, 23.25)	0.30	
Widowed/Divorced/Separated	1.25 (0.40, 3.95)	0.70	
Family PIR			0.31
≤1.3	1.31 (0.65, 2.65)	0.44	
1.3–3.5	1.91 (0.83, 4.39)	0.13	
>3.5	4.19 (1.38, 12.75)	0.01	
BMI ~ kg/m2			0.95
<25	0.80 (0.08, 7.58)	0.84	
≥25	1.63 (0.99, 2.70)	0.06	
Smoking behavior ~ %			<0.01
Former	4.82 (2.37, 9.80)	<0.01	
Never	0.96 (0.43, 2.12)	0.92	
Now	0.48 (0.08, 2.87)	0.41	
Hypertension ~ %			0.28
No	1.21 (0.40, 3.64)	0.72	
Yes	1.76 (0.97, 3.22)	0.06	
HbA1c control ~ %			0.76
High	1.92 (1.05, 3.49)	0.03	
Low	1.66 (0.68, 4.06)	0.26	
DN ~ %			0.74
No	1.83 (0.94, 3.58)	0.08	
Yes	1.75 (0.73, 4.20)	0.20	
Lipid control ~ %			0.03
High	1.15 (0.62, 2.13)	0.65	
Low	2.50 (1.31, 4.74)	0.01	

* Model was adjusted for age, sex, race, marital status, BMI, PIR, smoking habits, hypertension, lipid control, diabetic nephropathy, and HbA1C status. (except grouping variables).

**Table 4:** Results of subgroup analyses of PHQ-9 scores and risk of DR.

Subgroup variable	PHQ9-score*		
	OR (95% CI)	*p* value	*p* for interaction
Age			<0.01
<65	1.00 (0.96, 1.05)	0.84	
≥65	1.13 (1.07, 1.19)	<0.01	
Gender			0.09
Female	1.01 (0.95, 1.07)	0.80	
Male	1.11 (1.04, 1.18)	<0.01	
Race			0.33
Mexican American	1.05 (0.94, 1.18)	0.30	
Non-Hispanic Black	1.00 (0.92, 1.09)	0.92	
Non-Hispanic White	1.06 (1.01, 1.12)	0.02	
Other Hispanic	1.00 (0.86, 1.16)	0.99	
Other Race	1.05 (0.97, 1.13)	0.25	
Marital status			0.95
Married/Living with Partner	1.06 (1.01, 1.11)	0.02	
Never married	1.04 (0.86, 1.27)	0.65	
Widowed/Divorced/Separated	1.07 (0.99, 1.15)	0.08	
Family PIR			0.99
≤1.3	1.07 (1.02, 1.13)	0.01	
1.3–3.5	1.05 (0.99, 1.12)	0.10	
>3.5	1.08 (1.01, 1.16)	0.03	
BMI ~ kg/m2			0.02
<25	1.40 (1.11, 1.76)	0.01	
≥25	1.04 (1.00, 1.08)	0.03	
Smoking behavior ~ %			0.18
Former	1.14 (1.07, 1.22)	<0.01	
Never	1.03 (0.97, 1.09)	0.31	
Now	1.01 (0.91, 1.12)	0.86	
Hypertension ~ %			0.27
No	1.03 (0.95, 1.12)	0.46	
Yes	1.06 (1.01, 1.11)	0.01	
HbA1c control ~ %			0.98
High	1.06 (1.01, 1.11)	0.02	
Low	1.05 (0.99, 1.12)	0.09	
DN ~ %			0.86
No	1.06 (1.01, 1.11)	0.03	
Yes	1.08 (1.01, 1.14)	0.02	
Lipid control ~ %			0.01
High	1.01 (0.95, 1.06)	0.84	
Low	1.10 (1.05, 1.15)	<0.01	

* Model was adjusted for age, sex, race, marital status, BMI, PIR, smoking habits, hypertension, lipid control, diabetic nephropathy, and HbA1C status (except grouping variables).

## 4 Discussion

This study investigated the association between depression and the risk of diabetic DR in a representative US population using NHANES data. Our findings revealed that the weighted prevalence of DR in the included population was 18.91%. Several factors, including glycemic control (HbA1c), economic status (PIR), kidney function (albumin-to-creatinine ratio), and psychological health (depression prevalence), were significantly associated with DR. Importantly, depression was independently associated with an increased risk of DR, and this relationship persisted after adjusting for multiple covariates. Similarly, our RCS results also indicate a significant linear relationship. Subgroup analyses further highlighted the differential impact of depression on DR risk across specific demographic and clinical groups.

Our findings align with previous research that has demonstrated a link between depression and diabetes complications (19, 20), including DR. Studies have suggested that depression exacerbates diabetes management challenges, potentially worsening glycemic control and increasing the risk of vascular complications (21, 22). The observed association between depression and DR in our study corroborates these findings, highlighting the psychological burden as a significant risk factor.

Interestingly, we identified a nonlinear relationship between depression levels (measured by PHQ-9 scores) and DR risk in individuals with poor lipid control. This was determined using RCS models, which allow for a flexible exploration of dose–response relationships. By visualizing the RCS curves, we observed a significant departure from linearity, particularly in the subgroup with elevated lipid levels, suggesting that incremental increases in depression scores do not uniformly translate to higher DR risk. Such nonlinearity was less apparent in other metabolic subgroups, underscoring the complex interplay between depression and lipid metabolism in DR progression.

To clarify this further, we applied restricted cubic spline analysis with three knots at specific percentiles (10th, 50th, and 90th percentiles) of PHQ-9 scores. This choice was based on established statistical practices for adequately capturing potential nonlinear relationships while avoiding overfitting. The RCS plots supported our findings, revealing distinct trends in the depression-DR relationship across lipid control subgroups. This methodological detail strengthens the validity of our conclusions and underscores the importance of stratified analyses in future research.

The mechanisms underlying the observed associations are multifaceted. Depression may activate the hypothalamic–pituitary–adrenal (HPA) axis and promote systemic inflammation (23–25), which can exacerbate vascular dysfunction, a key contributor to DR development (26, 27). Chronic inflammatory states, driven by depression, may lead to increased production of pro-inflammatory cytokines, such as interleukin-6 and tumor necrosis factor-alpha (23), which are known to damage retinal microvasculature. Additionally, oxidative stress, a hallmark of both depression and diabetes (28, 29), may further accelerate retinal damage by disrupting endothelial function and promoting apoptosis in retinal cells (30, 31).

Behavioral factors may also play a significant role. Individuals with depression often experience reduced motivation for self-care (32), including adherence to medication, physical activity, and dietary recommendations. Such behaviors could lead to suboptimal glycemic and lipid control, compounding the risk of DR. The significant interaction between depression and lipid control observed in our study suggests that dyslipidemia may serve as a key mediator, amplifying inflammatory or oxidative stress pathways linking depression and DR. These findings suggest that addressing depression in diabetic patients could have benefits for metabolic and vascular health (33–35).

In addition, an emerging body of evidence suggests that religious activities may play a protective role in physical and mental health, including the management of chronic diseases. Religious engagement has been associated with reduced levels of stress, better psychological well-being, and improved adherence to medical treatments. Bungau and Popa discuss the historical and contemporary perspectives on the interplay between religion and health, underscoring how spiritual practices can promote healing and resilience (36). Applying these insights to diabetes care, religious involvement may positively influence patients’ mental health by offering social support, stress coping mechanisms, and a sense of purpose. Such factors may mitigate the psychological burden associated with diabetes, potentially reducing the risk of complications, including DR. Future research should examine the impact of religious participation on metabolic outcomes and investigate whether integrating faith-based approaches in diabetes management can provide psychological and physiological benefits.

The public health implications of our findings are substantial. Depression screening should become an integral part of routine care for diabetic patients, particularly in high-risk subgroups such as older adults, males, and those with well-controlled lipids. Targeted mental health interventions, including psychotherapy or pharmacological treatments, may mitigate the adverse effects of depression on DR risk. Furthermore, addressing socio-economic determinants, such as improving access to healthcare and financial support, may enhance both mental and physical health outcomes in this population.

In clinical practice, an integrated care model that combines psychological, metabolic, and social support is essential (37). The significant associations between lower PIR and DR prevalence emphasize the importance of policies aimed at reducing economic barriers to comprehensive diabetes care. Additionally, personalized management strategies that account for individual variability in depression’s impact on DR, such as those influenced by lipid control, could optimize patient outcomes.

Several limitations of this study should be acknowledged. First, the cross-sectional design precludes causal inferences between depression and DR. Longitudinal studies are necessary to determine whether depression directly contributes to DR development or if the relationship is bidirectional. Second, reliance on self-reported depression symptoms (PHQ-9 scores) may introduce reporting bias, and future studies should incorporate clinical diagnoses of depression for validation. Third, while we adjusted for multiple confounders, residual confounding from unmeasured variables, such as diet patterns or physical activity, cannot be excluded. For instance, adherence to healthy dietary patterns has been associated with better glycemic control and reduced inflammation, both of which are important in DR development. Future studies should incorporate comprehensive dietary assessments to provide a more nuanced understanding of the depression-DR relationship. Moreover, NHANES data represent a specific population, and the generalizability of these findings to other populations should be examined in future studies. Finally, in the diabetes interview questionnaire, the diagnosis of DR relied on participants’ self-reports. However, in the early stages of DR, patients may not perceive any symptoms, and medical examinations such as fluorescein fundus angiography (FFA) are required for detection. Only in the middle and late stages do complications such as blurred vision, visual distortion, and floating black shadows become apparent. This suggests that the use of self-reported measures may primarily capture cases of moderate to late-stage DR. We acknowledge this limitation and recommend that future studies explore more rigorous assessment methods to enhance diagnostic accuracy.

Future research should focus on conducting more rigorous longitudinal studies to elucidate the causal relationship between depression and DR and to evaluate the potential role of psychological interventions in reducing the long-term risk of DR. Additionally, mechanistic studies exploring the role of systemic inflammation, oxidative stress, and lipid metabolism in mediating the depression-DR association could provide valuable insights. Research should also focus on interventional strategies, such as cognitive-behavioral therapy, anti-inflammatory treatments, or lipid-modifying therapies, in reducing DR risk among depressed diabetic patients. Exploring the efficacy of combined interventions targeting both psychological and metabolic factors could pave the way for innovative, multi-faceted treatment approaches.

## 5 Conclusion

This study highlights the significant and independent association between depression and DR in a representative US population. These findings underscore the importance of addressing psychological health as part of comprehensive diabetes management. By integrating mental health care with traditional metabolic control strategies, we can potentially mitigate the burden of DR and improve the quality of life for individuals with diabetes. Furthermore, targeted interventions and personalized care models offer promising avenues for reducing the impact of depression on diabetes complications.

## Data Availability

Publicly available datasets were analyzed in this study. This data can be found here: https://wwwn.cdc.gov/nchs/nhanes/default.aspx.
